# Efficient Electromagnetic Wave Absorption and Thermal Infrared Stealth in PVTMS@MWCNT Nano-Aerogel via Abundant Nano-Sized Cavities and Attenuation Interfaces

**DOI:** 10.1007/s40820-023-01218-y

**Published:** 2023-11-17

**Authors:** Haoyu Ma, Maryam Fashandi, Zeineb Ben Rejeb, Xin Ming, Yingjun Liu, Pengjian Gong, Guangxian Li, Chul B. Park

**Affiliations:** 1 College of Polymer Science and Engineering, State Key Laboratory of Polymer Materials Engineering, Sichuan University, 24 Yihuan Road, Nanyiduan, Chengdu, 610065 Sichuan People’s Republic of China; 2 Microcellular Plastics Manufacturing Laboratory, Department of Mechanical and Industrial Engineering, University of Toronto, 5 King’s College Road, Toronto, ON M5S 3G8 Canada; 3 Jiangsu JITRI Advanced Polymer Materials Research Institute, Tengfei Building, 88 Jiangmiao Road, Jiangbei New District, Nanjing, 211800 Jiangsu People’s Republic of China; 4 MOE Key Laboratory of Macromolecular Synthesis and Functionalization, Department of Polymer Science and Engineering, International Research Center for X Polymers, Zhejiang University, 38 Zheda Road, Hangzhou, 310027 People’s Republic of China

**Keywords:** Nano-pore size, Heterogeneous interface, Electromagnetic wave absorption, Thermal infrared stealth, Nano-aerogel

## Abstract

Pre-polymerized vinyl trimethoxy silane (PVTMS)@MWCNT nano-aerogel system was constructed via radical polymerization, sol–gel transition and supercritical CO_2_ drying. The fabricated organic–inorganic hybrid PVTMS@MWCNT aerogel structure shows nano-pore size (30–40 nm), high specific surface area (559 m^2^ g^−1^), high void fraction (91.7%) and enhanced mechanical property: (1) the nano-pore size is beneficial for efficiently blocking thermal conduction and thermal convection via Knudsen effect (beneficial for infrared (IR) stealth); (2) the heterogeneous interface was beneficial for IR reflection (beneficial for IR stealth) and MWCNT polarization loss (beneficial for electromagnetic wave (EMW) attenuation); (3) the high void fraction was beneficial for enhancing thermal insulation (beneficial for IR stealth) and EMW impedance match (beneficial for EMW attenuation). Guided by the above theoretical design strategy, PVTMS@MWCNT nano-aerogel shows superior EMW absorption property (cover all *Ku*-band) and thermal IR stealth property (Δ*T* reached 60.7 °C). Followed by a facial combination of the above nano-aerogel with graphene film of high electrical conductivity, an extremely high electromagnetic interference shielding material (66.5 dB, 2.06 mm thickness) with superior absorption performance of an average absorption-to-reflection (*A/R*) coefficient ratio of 25.4 and a low reflection bandwidth of 4.1 GHz (*A/R* ratio more than 10) was experimentally obtained in this work.

**Supplementary Information:**

The online version contains supplementary material available at 10.1007/s40820-023-01218-y.

## Highlights

- PVTMS@MWCNT nano-aerogel with nano-pore size and abundant heterogeneous interface was fabricated via radical polymerization, sol–gel transition and CO_2_ drying.
- The nano-aerogel shows superior electromagnetic wave absorption property (RL_min_ = −36.1 dB and cover all *Ku*-band) and thermal infrared stealth property (Δ*T* reached 60.7 °C).
- Layered nano-aerogel/graphene film with high EMI shielding and absorption properties was obtained;

**Supplementary Information:**

The online version contains supplementary material available at 10.1007/s40820-023-01218-y.

## Graphical abstract

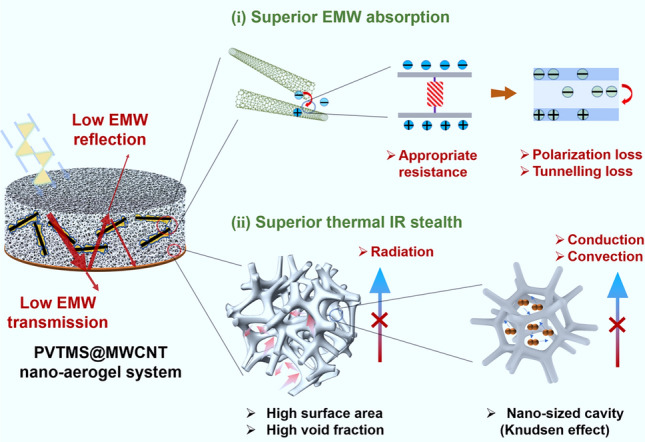

## Introduction

New generation materials with both superior electromagnetic wave (EMW) absorption property and infrared (IR) stealth property have received widespread attention due to their important application potentials, especially in the field of human health protection, precision instrument protection and modern military [1–3]. For example, in modern military area, materials with both IR stealth and EMW absorption property were beneficial for developing IR and EMW double stealth device. However, microwave absorbers need low reflectivity and high absorptivity, while IR stealth materials require high reflectivity and low IR absorptivity [3]. Thus, it seems to be challenging to integrate IR and EMW stealth in one material owing to the thoroughly opposite principles. Therefore, lots of effort have been made to prepare materials with both EMW absorption and IR stealth properties.

For EMW absorption materials, interfacial polarization loss [4, 5], conduction loss [6, 7] and multi-reflection loss [8] have been proven to be efficient methods to absorb the incident EMW. For example, Zhou et al*.* [9] fabricated multi-heterogeneous interface structure in CoNi/C aerogel, due to the enhanced interfacial polarization loss and the impedance match, hence achieved a minimum reflection loss value of –60.7 dB. Ma et al*.* [6] utilized conduction loss and polarization loss of graphene nanoribbon (GNR) structure in the cell wall of GNR/poly(vinylidene fluoride) nanocomposites foam, hence achieved high EMW absorption property (−54.1 dB). Xu et al*.* [8] fabricated reduced graphene oxide@ferroferric oxide (Fe_3_O_4_)/carbon nanotube/tetraneedle-like ZnO whisker@silver/waterborne polyurethane composite foams with aligned porous structures, hence achieved broadband microwave absorption performance (in the frequency range of 8.2–18.0 GHz) due to the multi-reflection of EMW and the progressive conductive structure design. However, it should be noted that the absorbed EMW energy via polarization loss, conduction loss and multi-reflection loss will be transformed into joule heat [10]. And if EMW energies are transformed into heat, the target surface temperature will increase and can be easily detected by IR detectors, hence deteriorating the IR stealth property [11].

Porous structure is beneficial for preparing superior IR stealth materials; because the high void fraction leads to decreased thermal conductivity and air/solid interface structure enhances IR reflection. Gu et al*.* [3] coated poly(3, 4-ethylenedioxythiophene):polystyrene sulfonate on melamine foam surface (with micrometer pore size) to create heterogeneous interface in porous structure, hence prepared materials with desired IR stealth property (Δ*T* reached to 35.9 °C). Wu et al*.* [12] used freeze-drying method to prepare porous reduced graphene oxide/Fe_3_O_4_ materials (with micrometer pore size) and the IR stealth property (Δ*T*) reached 34.8 °C. Therefore, porous materials (containing air phase) show great potential for preparing high-performance IR stealth materials. However, it should be also noted that solid skeleton in micrometer porous materials could be an idealized thermal conduction pathway, which will deteriorate the IR stealth property of materials [13].

Nano-aerogel materials (with nano-sized air distribution) could be used for more effectively blocking thermal conduction and IR signal due to the unique structure including nano-pore size, high specific surface area and high void fraction [14, 15]. For IR stealth materials, nano-pore size of aerogel materials will induce Knudsen effect and greatly restrict the free movement of air molecules, hence greatly decreasing the thermal conduction (even lower than air) and thermal convection of nano-aerogel materials [16, 17]; high specific interface area will be beneficial for enhancing the IR wave reflection and improving IR stealth property of materials according to the Stefan–Boltzmann theory [18]; High void fraction will be beneficial for decreasing the thermal conduction of materials and improving the EMW impedance match, hence beneficial for enhancing the IR stealth property and EMW absorption property, simultaneously.

In this work, pre-polymerized vinyl trimethoxy silane (PVTMS) with high cross-linking density was synthesized using the radical polymerization method, and then the spinodal decomposition method was used to prepare PVTMS@MWCNT/ethanol wet-gel. Then, supercritical CO_2_ (scCO_2_) drying method was used to prepare PVTMS@MWCNT nano-aerogel with high void fraction, high specific surface area and nano-pore size. It is found that the high void fraction was beneficial to enhance the EMW impedance match and the tailored MWCNT heterogeneous interface was beneficial to enhance EMW polarization loss (electron transporting) and electron tunneling loss of PVTMS@MWCNT nano-aerogel. Therefore, all-frequency absorption in *Ku*-band (12.4–18 GHz) could be achieved with an optimized MWCNT nanofiller structure. The EMW absorption property of obtained PVTMS@MWCNT nano-aerogel even reached −36.1 dB. Meanwhile, the high specific surface area was beneficial for enhancing the IR reflection and nano-pore size was beneficial for decreasing the thermal conductivity of PVTMS@MWCNT nano-aerogel, hence greatly enhancing the IR stealth property (Δ*T* reached 60.7 °C). Then, by simply combining this EMW absorption aerogel layer (PVTMS@MWCNT nano-aerogel) with an ultra-thin graphene film layer, a high EMI shielding material with excellent EMW absorption property (an average *A/R* ratio of 25.4 and EMW absorption bandwidth (EBW) of 4.1 GHz) was successfully fabricated in this work. Therefore, from both experimental and theoretical viewpoints, this work provides a guideline for nano-scale structural designation of porous materials in the application of EMW absorbing, EMI shielding and IR stealth.

## Experimental

### Materials

Di-tert-butyl peroxide (DTBP, 98%, thermal initiator) and vinyl trimethoxy silane (VTMS, 98%, silica precursor) were purchased from Sigma-Aldrich. Anhydrous ethanol (100%, solvent) and ammonium hydroxide solution (28–30%, ACS Grade, base catalyst) were purchased from GreenField Global and VWR, respectively. All the chemicals were used as received. Multi-walled carbon nanotubes (MWCNTs, diameter: 9.5 nm; length: 1.5 μm) were purchased from Nanocyl SA, Belgium (NC7000™). Carbon dioxide (purity ≥ 99%, Linde Gas) was used for scCO_2_ drying.

### Sample Preparation

#### Polymeric Precursor Synthesis

PVTMS was prepared by the method discussed in our previous studies [19, 20]. First, 6 g of DTBP was dissolved in 60 g of VTMS and poured into a three-neck flask equipped with a condenser and stirrer. The reaction was initiated under a nitrogen atmosphere at 150 °C with a stirring speed of 200 rpm. After 3 h, the flask was placed in a vacuum oven at a temperature of 150 °C to remove the unreacted monomer and initiator.

#### Sol–Gel Synthesis of Hybrid Aerogels

**Fig. 1:**
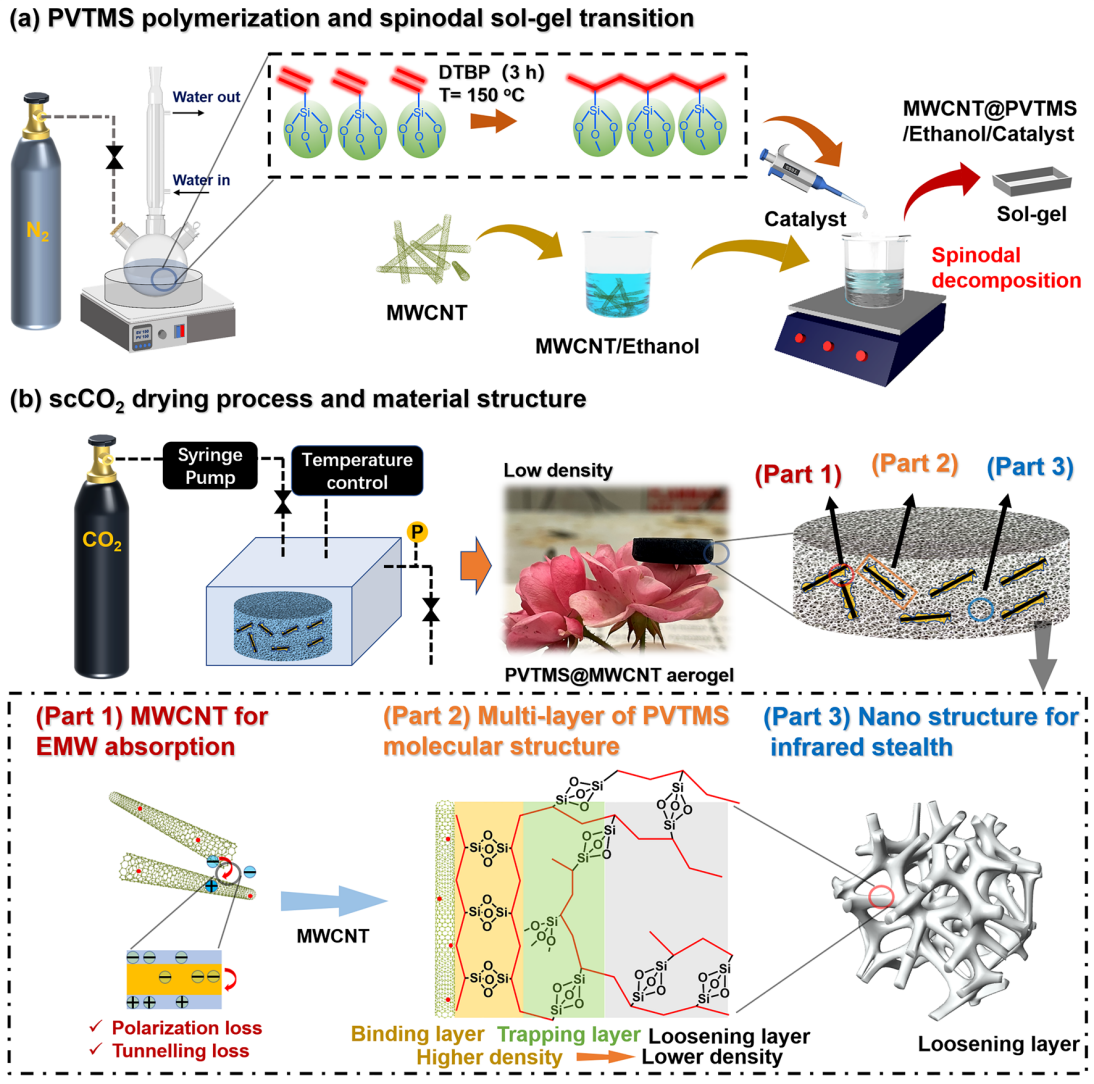
**a** Schematic illustration of PVTMS polymerization and sol–gel synthesis of PVTMS@MWCNT/ethanol wet gel; **b** scCO_2_ drying method to prepare PVTMS@MWCNT aerogel with MWCNT structure for EMW absorption (Part 1), mutilayer of PVTMS molecular structure (Part 2), nano structure for infrared stealth (Part 3)

As shown in Fig. 1a, 4 g PVTMS was dissolved in 20 mL ethanol and the solution was then stirred at 40 °C for 0.5 h. A certain amount of nanofiller was added to 20 mL ethanol solution followed by sonication for 0.5 h in a bath sonicator. The above two batches of ethanol solutions were then mixed and followed by a continuous stirring for another 0.5 h. After mixing, the base catalyst with water/Si molar ratio of 8 was added to the mixture for inducing spinodal decomposition. The mixture was further stirred for 1 more minute followed by transferring to the molds. The molds were kept in an oven at 40 °C for up to 12 h until gelation was completed. The wet gels were aged in ethanol for 24 h and, finally, went through solvent exchange with liquid CO_2_ at a pressure of 10.34 MPa (1500 psi), and dried with scCO_2_ at 45 °C.

#### Double-Layered PVTMS@MWCNT/Graphene Samples

Graphene films with superior electrical conductivity and EMW reflection property was prepared via solvent casting and thermal reduction [21–23], and the total thickness is around 0.03 mm. Then, PVTMS@MWCNT nanocomposite aerogel was combined with reflection layer (graphene film) using glue to prepare double layer aerogel/graphene EMI shielding materials.

### Characterization

Transmission electron microscope (TEM, Talos F200i) was used to study the microstructure of the aerogels. X-ray photoelectron spectroscope (XPS, Thermo Scientific K-Alpha) was used to study the element content of pristine PVTMS, PVTMS@MWCNT aerogel. The total thermal conductivity of the aerogel samples was measured using a Hot Disk TPS2500S thermal constants analyzer. Pore size distribution and the surface area of PVTMS@MWCNT aerogel were also measured by the Brunauer–Emmett–Teller (BET) test using an Autosorb IQ (Quantachrome Instruments). Infrared thermal imaging photos with the information of different sample’ (thickness: 5.6 mm) surface temperature and infrared stealth property were taken by an infrared imaging device (Fluke-Ti32S).

PNA-X network analyzer (Keysight N5232B) was used to evaluate EMW absorption property (single layer) and EMI shielding property of samples [24, 25]. According to waveguide method, real and imaginary parts of permittivity (*ε′* and *ε″*) and permeability (*μ′* and *μ″*) of PVTMS@MWCNT aerogel in *Ku*-band frequency range (12.4–18 GHz) were tested. EMW absorption performance (Reflection Loss, RL) and impedance match (*Z*_in_*/Z*_0_) can be calculated by the measured complex permittivity and permeability using the following equations [26, 27]:

1 $${\text{RL}} = 20\log \frac{{\left| {Z_{{{\text{in}}}} - Z_{0} } \right|}}{{\left| {Z_{in} + Z_{0} } \right|}}$$

2 $$Z_{{{\text{in}}}} = Z_{0} \sqrt {\frac{{\mu_{r} }}{{\varepsilon_{r} }}} \tanh \left( {j\left( {\frac{2\pi }{c}} \right)\sqrt {\mu_{r} \varepsilon_{r} } fd} \right)$$

where *Z*_in_ is the input impedance of the material (Ω) and *Z*_0_ refers to the impedance of free space (normally 377 Ω). *μ*_*r*_ and *ε*_*r*_ refer to the complex permeability and permittivity of the material, respectively. *f* and *d* represent the applied EMW frequency (Hz) and the thickness (m) of the material. *c* is the speed of light (m/s).

To determine the EMW attenuation capability of the material, the attenuation constant *α* can be evaluated by the equation:

3 $$\alpha = \left( {\frac{\sqrt 2 \pi f}{c}} \right)\sqrt {\mu^{\prime\prime}\varepsilon^{\prime\prime} - \mu^{\prime}\varepsilon^{\prime} + \sqrt {\left( {\mu^{\prime\prime}\varepsilon^{\prime\prime} - \mu^{\prime}\varepsilon^{\prime}} \right)^{2} + \left( {\mu^{\prime}\varepsilon^{\prime\prime} + \mu^{\prime\prime}\varepsilon^{\prime}} \right)^{2} } }$$

The scattering parameters, S_11_ and S_21_, were used to calculate the total SE (SE_T_), reflection SE (SE_R_), and absorption SE (SE_A_). *R*, *T* and *A* coefficients were also calculated from the scattering parameters, *S*_11_ and *S*_21_ [28, 29].

## Results and Discussion

### PVTMS@MWCNT Aerogel Preparation and the Corresponding Structure Characterization

As Fig. 1a shown, PVTMS polymer chain with high inorganic cross-linking density was prepared using radical polymerization method. The high cross-linking density was beneficial for fabricating aerogel with nano-pore size and high surface area, and the hybrid organic–inorganic molecular structure was beneficial for enhancing mechanical property of nano-aerogel materials [30, 31]. Then, spinodal decomposition method was used to prepare PVTMS@MWCNT wet gel (Fig. 1a), through this method, sol–gel transition speed could be greatly enhanced [32, 33]. Finally, in order to achieve nano-pore structure and high specific surface area, scCO_2_ drying method was used to prepare PVTMS@MWCNT nano-aerogel (Fig. 1b) [19]. The fabricated PVTMS@MWCNT nano-aerogel with abundant heterogeneous interface (Part 1), multilayer of PVTMS molecular structure (Part 2) and nano-pore size structure (Part 3) was beneficial for enhancing EMW absorption property, mechanical property and IR stealth property.

**Fig. 2:**
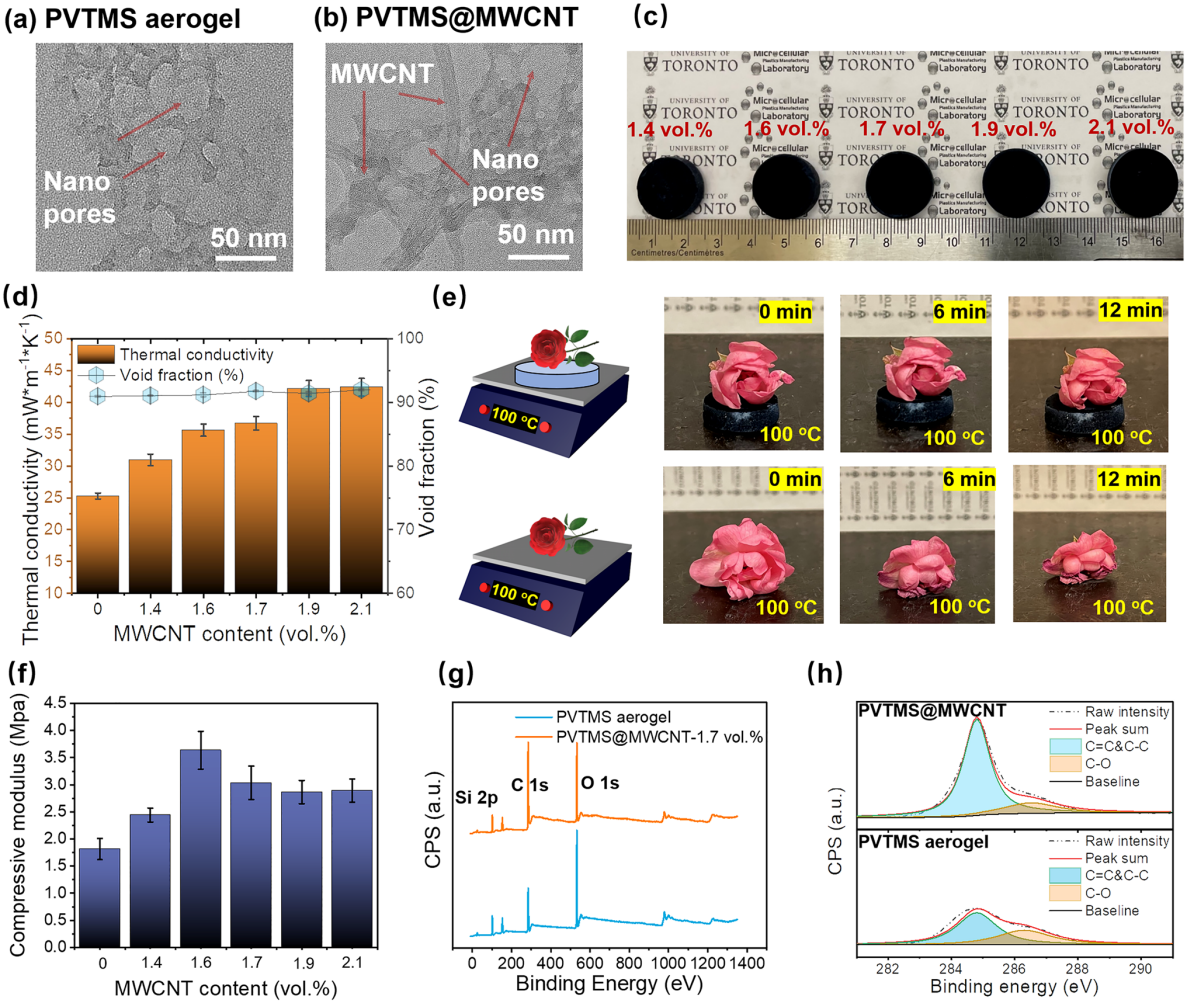
TEM micrographs of **a** pristine PVTMS aerogel and **b** PVTMS@MWCNT aerogel; **c** Digital micrograph of PVTMS@MWCNT aerogel with various nanofiller content; **d** Thermal conductivity and void fraction of PVTMS@MWCNT aerogel with various nanofiller content; **e** Thermal blocking property of PVTMS@MWCNT aerogel; **f** Compressive modulus of PVTMS@MWCNT aerogel with various nanofiller content; XPS spectral of pristine PVTMS aerogel and PVTMS@MWCNT aerogel at **g** 0–1400 eV and** h** 281–291 eV

Figure 2a, b shows TEM micrographs of pristine PVTMS nano-aerogel and PVTMS@MWCNT nano-aerogel, and they all show nano-pore size structure. Figure 2c shows digital micrograph of PVTMS@MWCNT aerogel with various nanofiller content, it is noted that the degree of aerogel shrinkage decreased with increasing MWCNT content after scCO_2_ drying (Fig. S3). For aerogel with nano-pore size, the capillary force in solvent exchange process is the main reason for aerogel shrinkage. As Fig. 1 (Part 2) and Fig. S2 show, PVTMS molecular chains could be absorbed on MWCNTs’ surface due to hydrogen bonding effect and Van der Waals forces adsorption effect [34–36]. Then, the high-density PVTMS aerogel layer could be generated around MWCNT during sol–gel transition process, and the high-density layer around MWCNT network will be beneficial for enhancing the solid skeleton strength and decreasing the shrinkage ratio during scCO_2_ drying process.

Figure 2d shows thermal conductivity and void fraction of PVTMS@MWCNT nano-aerogel with various MWCNT contents. The void fraction slightly increased from 90.9 to 92% with increasing MWCNT content, and the thermal conductivity also increased from 25.3 to 42.4 mW m^−1^ K^−1^ with increasing MWCNT content. The increased void fraction could be ascribed to the decreased shrinkage ratio due to the enhanced solid skeleton strength around MWCNTs; the increased thermal conductivity could be ascribed to the high thermal conductivity of MWCNTs. However, due to the high void fraction (large amount of air) and nano-pore size (Knudsen effect), PVTMS@MWCNT nano-aerogel still shows low thermal conductivity. Figure 2e presents that the low thermal conductivity PVTMS@MWCNT nano-aerogel could be used to effectively block the heat transfer and hence suppress the water evaporation process of the flower on the nano-aerogel upper surface. Figure 2f shows the compressive mechanical property of PVTMS@MWCNT nano-aerogel with various MWCNT content, and the mechanical property increased by adding MWCNTs to form solid absorption layer (Fig. 1(Part 2)). Figure 2g, h shows overall XPS spectral and C 1*s* XPS spectral of pristine PVTMS aerogel and PVTMS@MWCNT aerogel. It is noted that the C 1*s* characteristic peak at 284.8 eV greatly increased, which can be ascribed to the added MWCNTs (C=C or C–C). The increased carbon nanofiller is then beneficial for the construction of EMW absorption structure to effectively absorb the incident EMW.

### EMW Absorption Property of PVTMS@MWCNT Aerogel

**Fig. 3:**
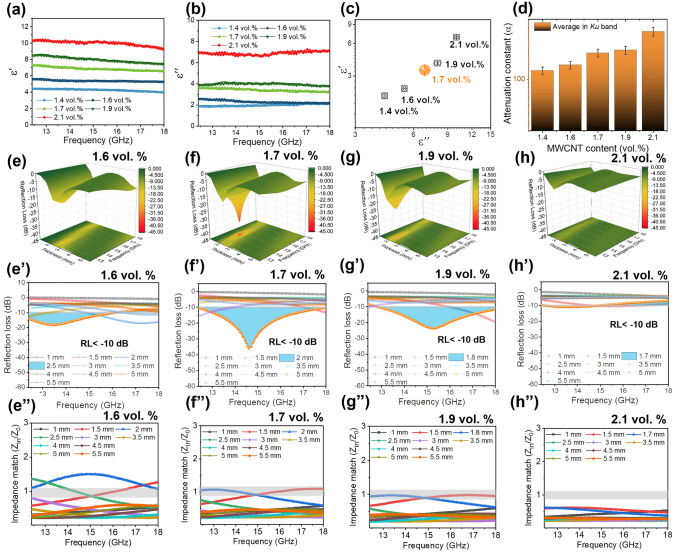
**a** Real part and **b** imaginary part of dielectric property of PVTMS@MWCNT nano-aerogel with various MWCNT contents; **c** Average real part and imaginary part of dielectric property of PVTMS@MWCNT aerogel in *Ku*-band; **d** Calculated attenuation constant of PVTMS@MWCNT aerogel with various nanofiller content; **e–h** 3D EMW absorption curves, **e’**–**h’** 2D EMW absorption curves and **e’’**–**h’’**impedance match (*Z*_in_*/Z*_0_) of PVTMS@MWCNT aerogel with various MWCNT contents

Figure 3a, b shows electromagnetic parameters (permittivity) of PVTMS@MWCNT nano-aerogel with various nanofiller content. The average electromagnetic parameters are presented in Fig. 3c, it is noted that the real part (*ε′*) and imaginary part (*ε″*) of dielectric permittivity all increased with increasing nanofiller content. The increased dielectric permittivity could be ascribed to the increased MWCNT content, and the increased dielectric loss could be ascribed to the increased conduction loss in nanocomposites with increasing MWCNT content [37, 38]. Figure 3d shows the attenuation constant (*α*) of PVTMS@MWCNT nano-aerogel with various nanofiller contents. The attenuation constant increases with increasing nanofiller content, which is ascribed to the conduction loss of MWCNTs.

Figure 3e–h and e’–h’ shows the EMW absorption property of PVTMS@MWCNT nano-aerogel with various MWCNT contents in both 3D and 2D. It is noted that the EMW absorption property of PVTMS@MWCNT nano-aerogel increased first and then decreased with increasing nanofiller content. Figure 3e”–h” shows the impedance match of PVTMS@MWCNT nano-aerogel at various thickness. It is noted that impedance match (*Z*_in_*/Z*_0_) of aerogel material gradually decreases with increasing MWCNT content. As Fig. 3f’’ shows, the impedance match of PVTMS@MWCNT nano-aerogel reached to 1, which means the lower reflection ratio of EMW at air/material interface. And the decreased reflection ratio will be beneficial for EMW absorption [39]. Hence, as Fig. 3f, f’ shown, the best EMW absorption property (all-*Ku* band, RL_min_ = –36.1 dB, 2 mm in thickness) was achieved by tailoring the MWCNT structure in PVTMS nano-aerogel system. As Figs. S7 and S8 show, the superior EMW absorption property could be ascribed to heterogeneous interface polarization loss and tunnelling loss.

Meanwhile, it is also noted that optimal EMW absorption thickness gradually decreases with increasing MWCNT content, this could be ascribed to the following two reasons: (1) the optimal impedance thickness gradually decreases with increasing MWCNT content; (2) The optimal EMW absorption thickness (T) could be expressed as follows [40]:

4 $${\text{T}} = n\frac{\lambda }{4} = nc/\left( {4f\sqrt {\left| {\mu_{r} } \right|\left| {\varepsilon_{r} } \right|} } \right)(n = 1,3,5......)$$

where* c* is the speed of light, *μ*_r_ and *ε*_r_ refer to the complex permeability and permittivity of the material, respectively. *f* represents the applied EMW frequency. Therefore, the optimal EMW absorption thickness gradually decreases with increasing dielectric property (Fig. 3a).

### IR Stealth Property of PVTMS@MWCNT Aerogel

**Fig. 4:**
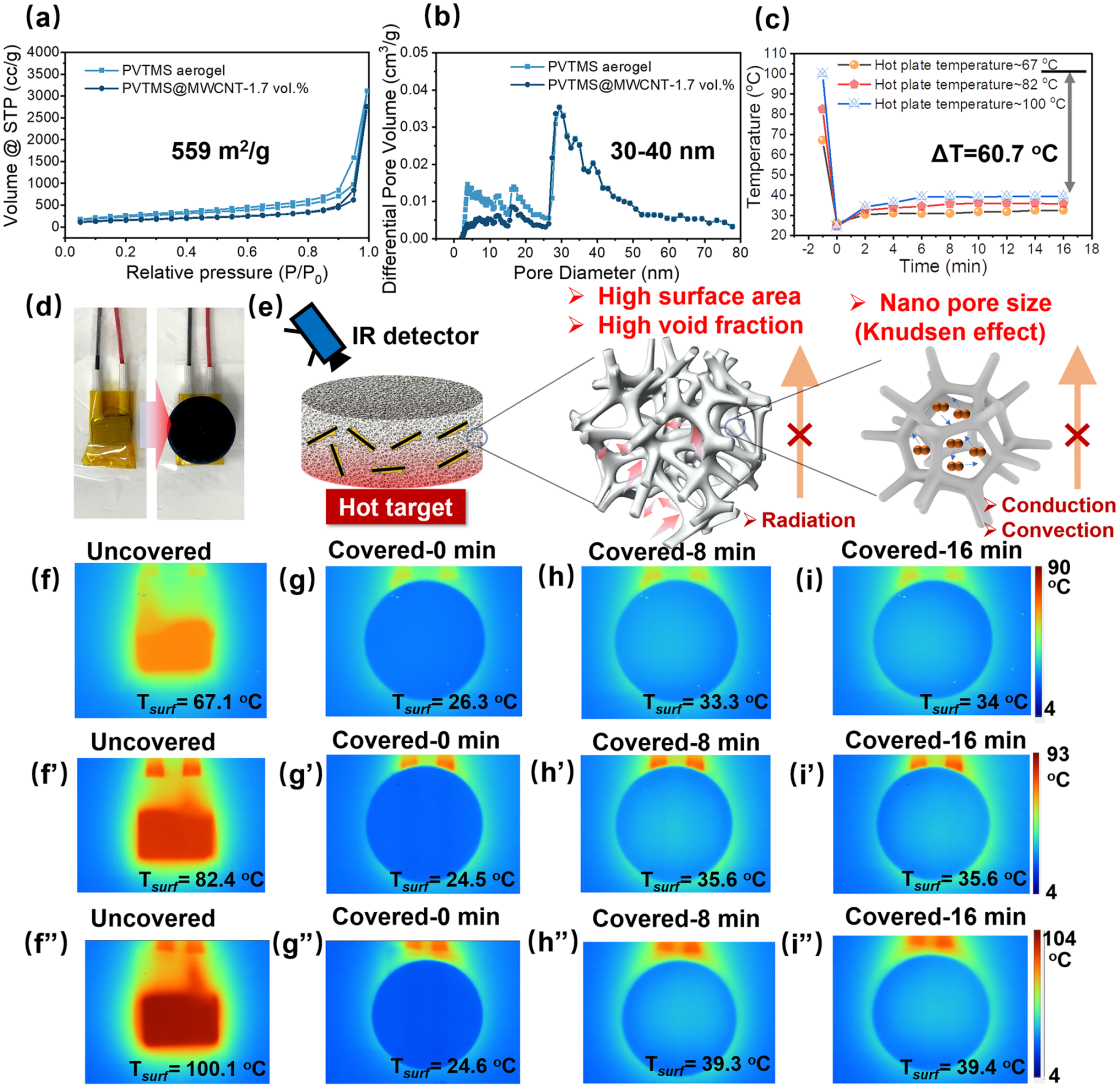
**a** Nitrogen adsorption–desorption isotherms and **b** pore diameter distribution curves of pristine PVTMS aerogel and PVTMS@MWCNT aerogel; **c** Variation tendency of the temperature detected on the upper surface of samples *versus* heating time at a fixed setting temperature; **d** digital micrograph of heat target uncovered and covered with PVTMS@MWCNT aerogel; **e** Infrared stealth mechanism of PVTMS@MWCNT aerogel; Thermal infrared images of PVTMS@MWCNT sample captured at intervals of 8 min from 0 to 16 min (**f**–**i** at setting *T* of 67.1 °C, **f’**–**i’** at setting *T* of 82.4 °C and **f’’**–**i’’** at setting *T* of 100.1 °C)

Figure 4 shows nitrogen adsorption–desorption isotherms of pristine PVTMS aerogel and PVTMS@MWCNT aerogel. The pore shapes and types were investigated based on IUPAC’s technical report on the physisorption of gases [41]. It can be observed that all samples exhibit type IV isotherm, which is a characteristic of mesoporous materials [41]. The hysteresis loop is generated due to the capillary condensation in mesopores with a diameter over 4 nm. Nitrogen adsorption–desorption analysis was also used to measure the specific surface area and pore size of the samples [20]. As Fig. 4a, b shows, PVTMS@MWCNT aerogel shows high specific surface area (559 m^2^ g^−1^) and nano pore size (30–40 nm).

In order to characterize the infrared stealth property of our PVTMS@MWCNT nano-aerogel, samples were placed on a heating platform at various temperatures (67.1, 82.4 and 100.1 °C) and thermal infrared images were captured. Figure 4f–i” shows thermal infrared images of PVTMS@MWCNT nano-aerogel captured at intervals of 8 min from 0 to 16 min at various heat source temperature including 67.1, 82.4 and 100.1 °C. The detailed upper surface temperature is presented in Fig. 4c, it is noted that the target signal was greatly decreased by covering PVTMS@MWCNT nano-aerogel on the hot plate, and the Δ*T* reached to 60.7 °C.

For thermal IR steal materials, low IR emissivity and low thermal conductivity are key factors for enhancing IR stealth property. As Fig. S11 shows, it is noted that PVTMS@MWCNT samples shows high infrared emissivity (0.95 at 3–5 µm and 0.94 at 8–14 µm). The proposed reason for the high infrared emissivity could be ascribed to the added MWCNT, which could act as black body, hence, to absorb and emit IR signal [16]. Figures 4e and S12 shows the IR stealth mechanism of PVTMS@MWCNT nano-aerogel. Generally, heat transfer mechanism including thermal radiation, thermal conduction and thermal convection. For PVTMS@MWCNT nano-aerogel, the high specific surface area and high void fraction were beneficial for blocking thermal radiation by reflectance and decreasing solid-phase thermal conduction, respectively; the nano-pore size was beneficial for blocking gas-phase thermal conduction and thermal convection via Knudsen effect. Hence, PVTMS@MWCNT nano-aerogel shows superior IR stealth property.

### Double-Layered PVTMS@MWCNT/Graphene Film for EMI Shielding

**Fig. 5:**
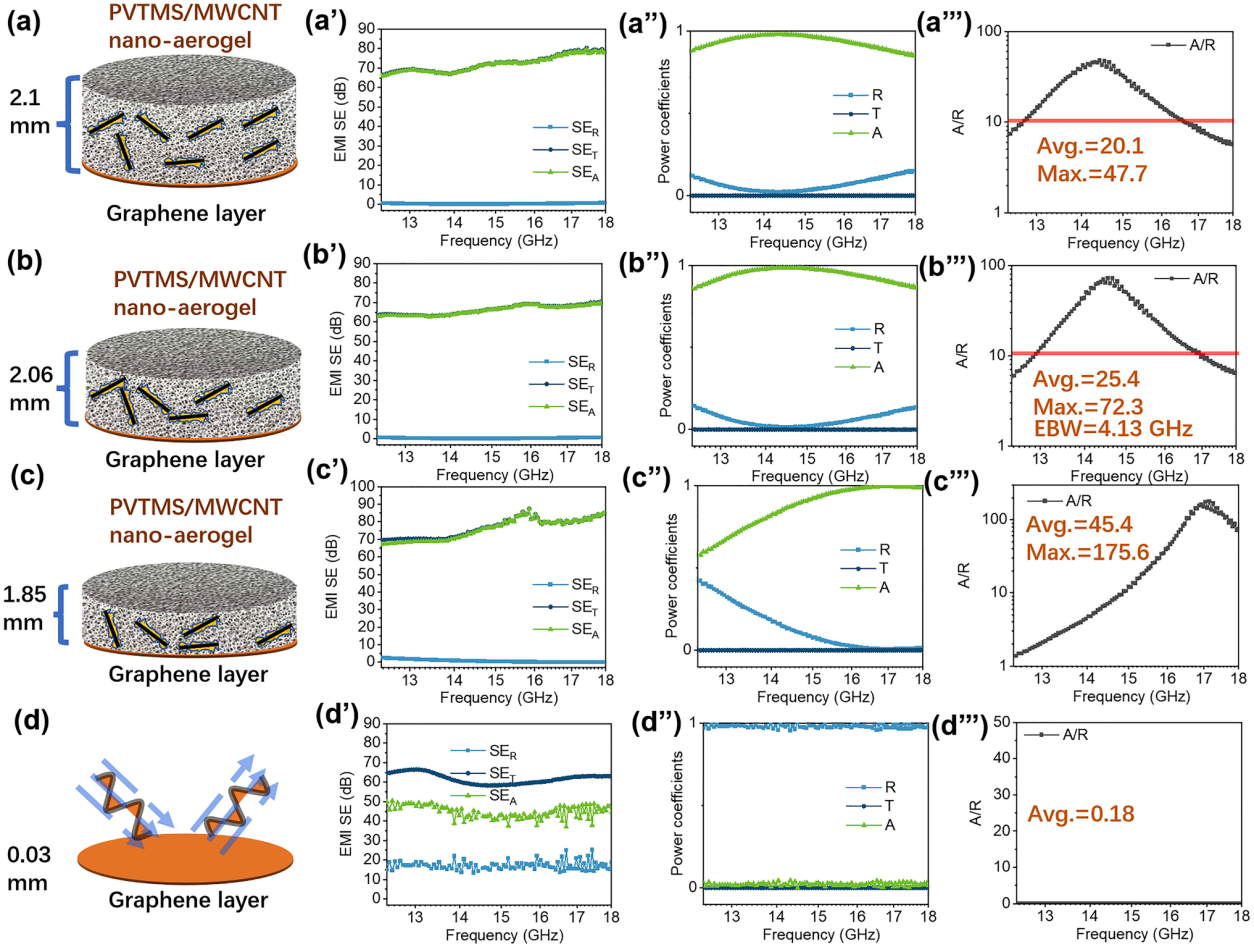
**a–d** Schematic illustration of double-layered EMI shielding materials combining PVTMS@MWCNT nano-aerogel (top layer) and graphene film layer (bottom layer); **a’**–**d’** EMI shielding effectiveness (*SE*) of double-layered samples with various nano-aerogel layer thickness, and **a’’**–**d’’** the corresponding reflection (*R*), transmission (*T*) and absorption values together **a’’’**–**d’’’** with *A/R* ratio

PVTMS@MWCNT aerogel also shows great potential for preparing double-layered material with superior EMI shielding property (absorption dominant). Hence, in this work, graphene film with high electrical conductivity and high EMW reflection property was used as the EMW reflection layer (bottom film layer) to construct a double-layered EMI shielding material with EMW absorption aerogel layer on top, as shown in Fig. 5. For pristine graphene film layer shown in Fig. 5d, d’, d’’ and d’’’, most incident EMW was reflected at the interface between air/graphene film (average *R* = 0.98) due to the film’s very high electrical conductivity. However, for those double-layered EMI shielding material, the reflection (SE_R_) greatly decreased and almost all of total EMI shielding (SE_T_) were from absorption (SE_A_). As shown in Fig. 5b’’, b’’’, the double-layered absorption material of ~ 2 mm in thickness with a reflection film shows the best EMW absorption property for EMI shielding application. The maximum *A/R* ratio reached to 72.3 and the average *A/R* ratio in *Ku* band was 25.4. The efficient EMW absorption bandwidth (EBW, *A/R* > 10) was 4.1 GHz.

**Fig. 6:**
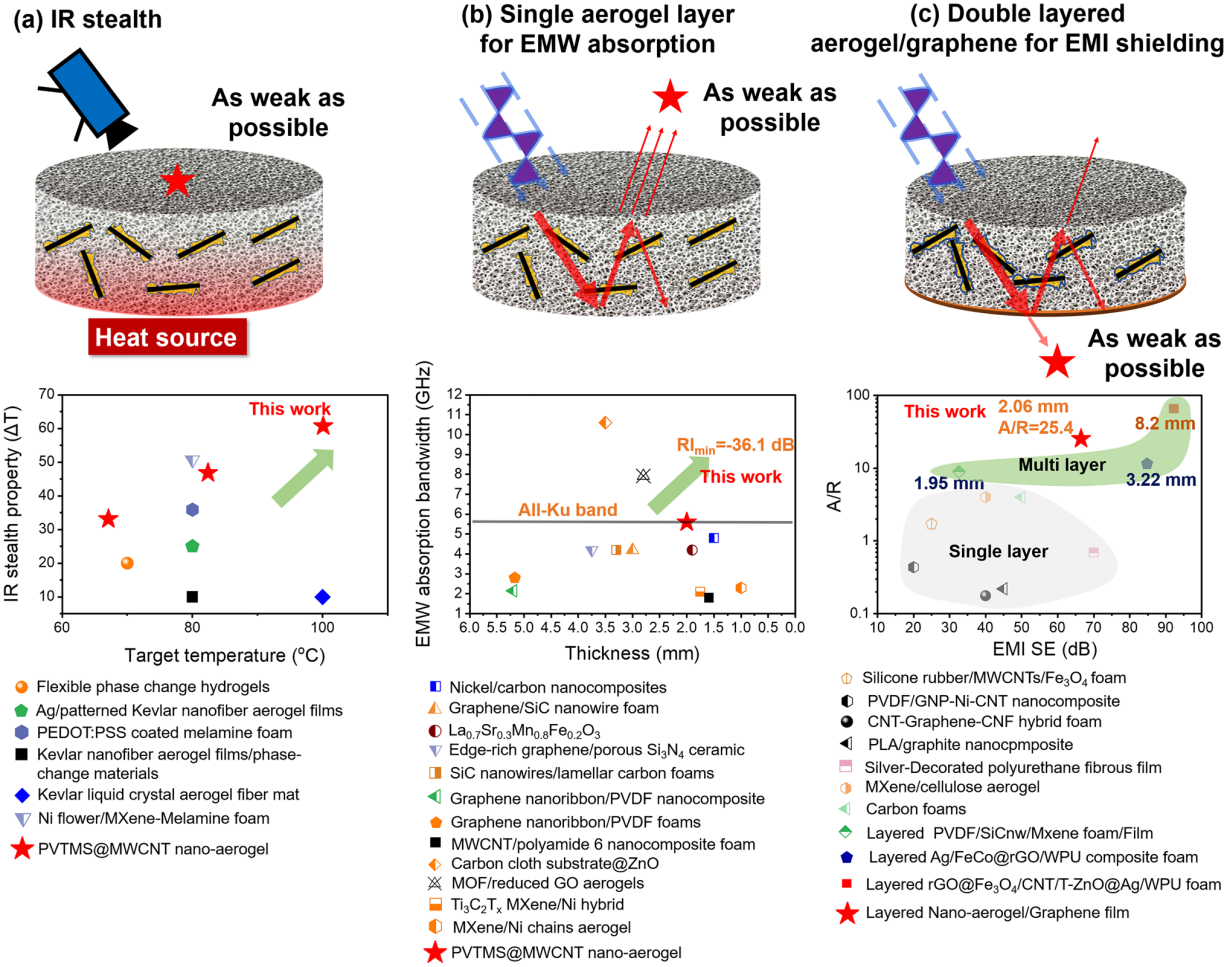
**a** Infrared stealth property [2, 3, 42–45] and **b** EMW absorption property [6, 7, 46–54] of PVTMS@MWCNT aerogel compared with references; **c** EMI shielding property of double-layered PVTMS@MWCNT aerogel/graphene film compared with references [8, 55–63]

Figure 6 summaries a series of studies on IR stealth (a), EMW absorption (b) and EMI shielding (c), it is noted that the fabricated PVTMS@MWCNT nano-aerogel shows superior IR stealth property, EMW absorption property and EMI shielding property, simultaneously. The main reason for this superior property could be ascribed to the tailored nano-sized cavities and abundant heterogeneous interface fabricated in PVTMS@MWCNT nano-aerogel system.

## Conclusion

In this work, MWCNT network structure with superior EMW absorption property was constructed in pre-polymerized vinyl trimethoxy silane (PVTMS) nano-aerogel system. Supercritical CO_2_ drying technology and spinodal decomposition method were used to prepare organic–inorganic hybrid PVTMS aerogel structure with nano-pore size, high specific surface area, high void fraction and enhanced mechanical property: (1) the nano pore size is beneficial for efficiently blocking thermal conduction and thermal convection; (2) the abundant heterogeneous interface was beneficial for IR refection and EMW absorption; (3) the high void fraction was beneficial for enhancing EMW impedance match of samples; Guided by the above theoretical design strategy, EMW absorbing PVTMS@MWCNT nano-aerogel with a -36.1 dB absorption performance cover all *Ku*-band (12.4–18 GHz) was successfully fabricated using the tailored structure design; and the PVTMS@MWCNT nano-aerogel shows superior IR stealth property (Δ*T* reached to 60.7 °C). Followed by a facial combination of the above nano-aerogel with graphene film of high electrical conductivity, an extremely high EMI shielding material (66.5 dB, 2.06 mm thickness) with superior absorption performance of an average absorption-to-reflection (*A/R*) ratio of 25.4 and a low refection bandwidth of 4.1 GHz (*A/R* ratio more than 10) was experimentally obtained in this work.

## Supplementary Information

Below is the link to the electronic supplementary material.

Supplementary file1 (PDF 1481 KB)
