# Supplementary material for: Efficient Electromagnetic Wave Absorption and Thermal Infrared Stealth in PVTMS@MWCNT Nano-Aerogel via Abundant Nano-Sized Cavities and Attenuation Interfaces
Source: Nanomicro Lett. 2023 Nov 17;16:20. doi: 10.1007/s40820-023-01218-y (PMC10656378; doi:10.1007/s40820-023-01218-y)
Supplement: Supplementary file 1 — Supplementary file1 (PDF 1481 KB) [file 40820_2023_1218_MOESM1_ESM.pdf]

Supporting Information for

Efficient Electromagnetic Wave Absorption and Thermal Infrared Stealth in PVTMS@MWCNT Nano-Aerogel *via* Abundant Nano-Sized Cavities and Attenuation Interfaces

Haoyu Ma[1,2,3], Maryam Fashandi[2], Zeineb Ben Rejeb[2], Xin Ming[4], Yingjun Liu[4], Pengjian Gong[1,*], Guangxian Li[1], and Chul B. Park[1,2,*]

[1]College of Polymer Science and Engineering, State Key Laboratory of Polymer Materials Engineering, Sichuan University, 24 Yihuan Road, Nanyiduan, Chengdu, Sichuan, People's Republic of China, 610065

[2]Microcellular Plastics Manufacturing Laboratory, Department of Mechanical and Industrial Engineering, University of Toronto, 5 King's College Road, Toronto, Ontario, Canada, M5S 3G8

[3]Jiangsu JITRI Advanced Polymer Materials Research Institute, Tengfei Building, 88 Jiangmiao Road, Jiangbei New District, Nanjing, Jiangsu, People's Republic of China, 211800

[4]MOE Key Laboratory of Macromolecular Synthesis and Functionalization, Department of Polymer Science and Engineering, International Research Center for X Polymers, Zhejiang University, 38 Zheda Road, Hangzhou 310027, People's Republic of China

* Corresponding authors. E-mail: park@mie.utoronto.ca (Chul B. Park), pgong@scu.edu.cn (Pengjian Gong)

S1 Results

S1.1 Material Structure

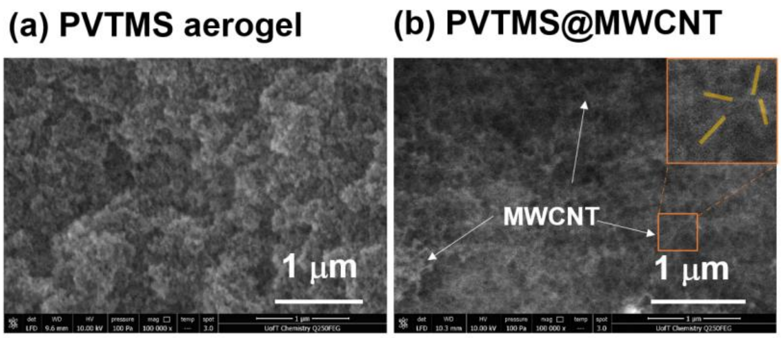

Fig. S1 SEM micrographs of pristine PVTMS aerogel (**a**) and PVTMS@MWCNT aerogel (**b**)

Scanning electron microscope (SEM, FEI, Quanta FEG 250) was used to study the microstructure of the aerogels.

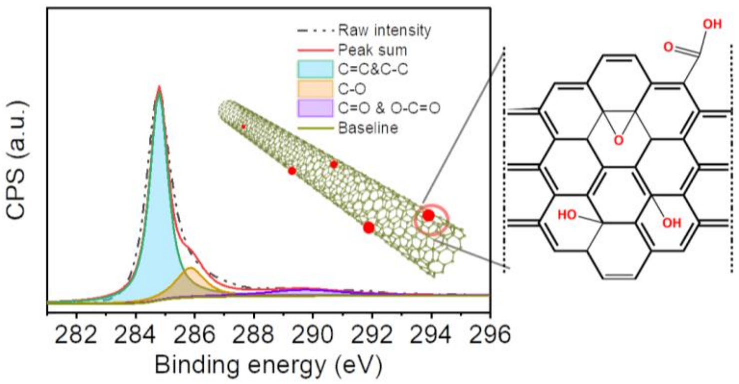

Fig. S2 C 1s XPS spectral of pristine MWCNT

Fig. **S2** shows the XPS spectral of pristine MWCNT used in this work, it is noted that there are some oxygen groups (-OH or -COOH) on the nanofiller surface. Therefore, hydrogen bonding structure could be formed between MWCNT (with -OH or -COOH) and PVTMS (with -Si-OH). The hydrogen bonding was beneficial for enhancing the molecular absorption effect of PVTMS on MWCNT surface.

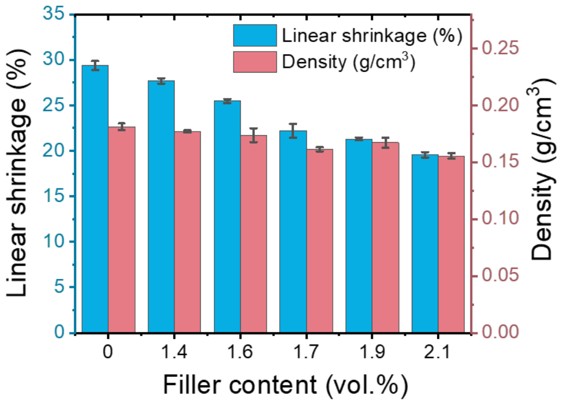

Fig. S3 Linear shrinkage and density of PVTMS@MWCNT aerogel with various MWCNT content

Fig. **S3** shows linear shrinkage and density of PVTMS@MWCNT aerogel with various nanofiller content, it is noted that the degree of aerogel shrinkage decreased with increasing MWCNT content after $scCO_2$ drying. Meanwhile, the density of nano-aerogel also slightly decreased with increasing MWCNTs nanofiller content.

S1.2 Mechanical Property of PVTMS@MWCNT Aerogel

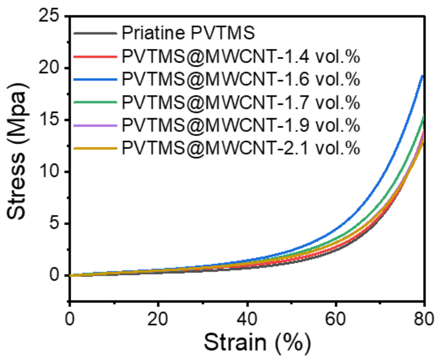

Fig. S4 Stress-strain curve of PVTMS@MWCNT aerogel with various MWCNT content

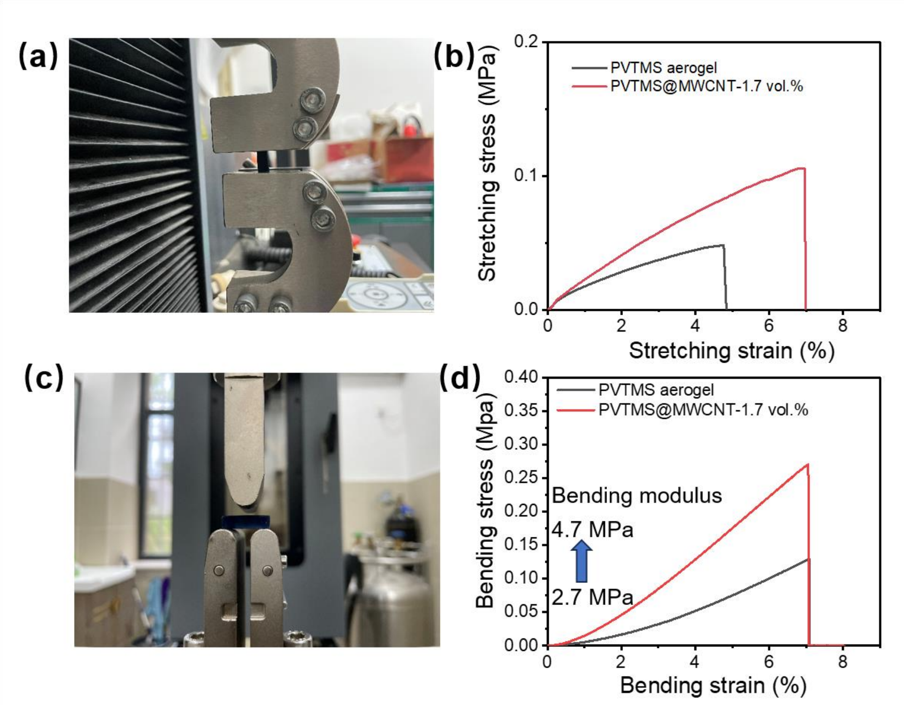

Fig. S5 Digital photos for stretching mechanical property test (**a**), stretching stress-strain curves of samples (**b**), Digital photos for bending mechanical property test (**a**), bending stress-strain curves of samples (**b**)

As Fig. S5a, b shows, comparing with pristine PVTMS aerogel, PVTMS@MWCNT aerogel shows enhanced tensile strength and stretching ratio at break. As **Fig.** S5c, d shows, the bending modulus of PVTMS@MWCNT aerogel increased from 2.7 MPa to 4.7 MPa by adding MWCNTs.

S1.3 EMW Absorption Property of PVTMS@MWCNT Aerogel

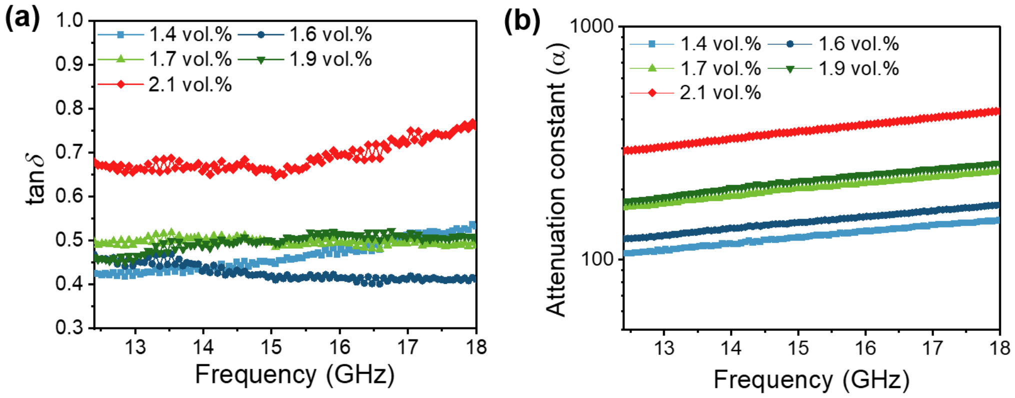

Fig. S6 Dielectric loss tangent (**a**) and attenuation constant (**b**) of PVTMS@MWCNT aerogel with various nanofiller content

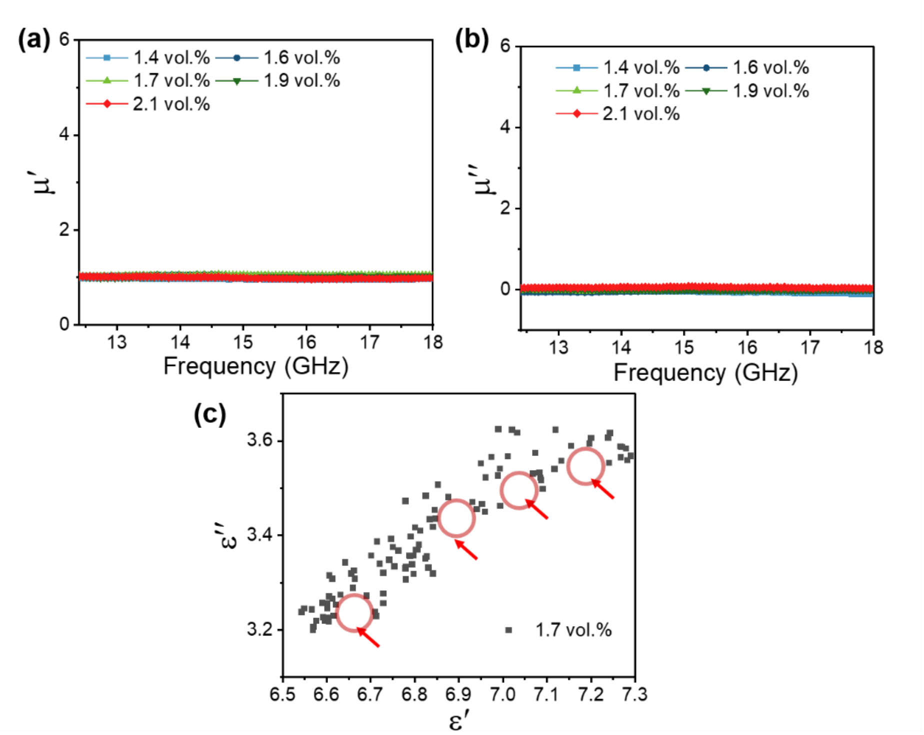

Fig. S7 Real part (**a**) and imaginary part (**b**) of permeability of PVTMS@MWCNT nano-aerogel with various MWCNT contents; Cole-Cole plots of PVTMS@ MWCNT-1.7 vol.% (**c**)

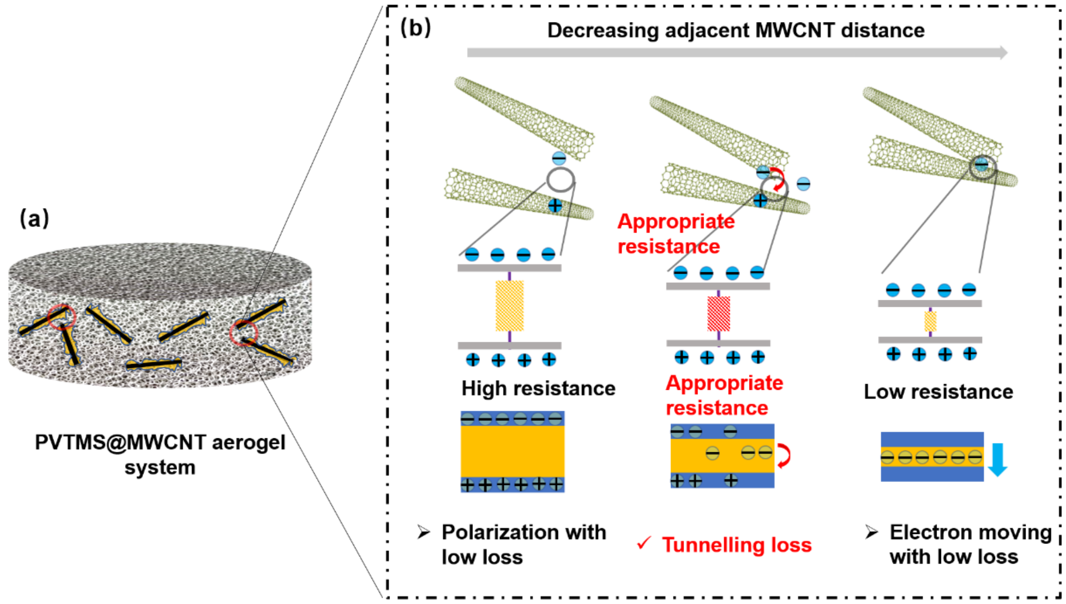

Fig. S8 Electrical network of PVTMS@MWCNT aerogel nanocomposites (**a**); Micro-scale electrical resistance model of carbon-PVTMS-carbon structure (**b**)

Besides polarization loss, tunnelling loss should be another important mechanism for PVTMS@MWCNT aerogel EMW absorption.

Fig. **S8** shows the conductive MWCNT structure in PVTMS aerogel system. At this carbon-PVTMS-carbon model, the contact resistance could be calculated based on the following equation [S1, S2]:

$$R_J = \frac{V}{A \times J} = \frac{d_{\min} \times P^2}{A \times e^2 \times \sqrt{2m\lambda}} \times \exp^{\left(\frac{4\pi \times d_{\min}}{P} \times \sqrt{2m\lambda}\right)} \quad \text{(S1)}$$

where V is electric potential difference (V), A is tunnel cross-sectional area (m^2), J is tunnel current density (A/m^2), d_{min} is carbon nanofiller minimum physical distance, P is Planck constant (N·m·s), m is electron mass (kg), λ is barrier height (eV), e is elementary charge (C).

It is noted that carbon nanofiller contact resistance positively correlated with physical distance (d_{min}). Therefore, the contact resistance will decrease with decreasing nanofiller distance. And when the d_{min} is at a suitable range, electrons can move between adjacent MWCNTs with high loss. At this situation, the EMW energy will be transferred into joule heat via tunnelling loss.

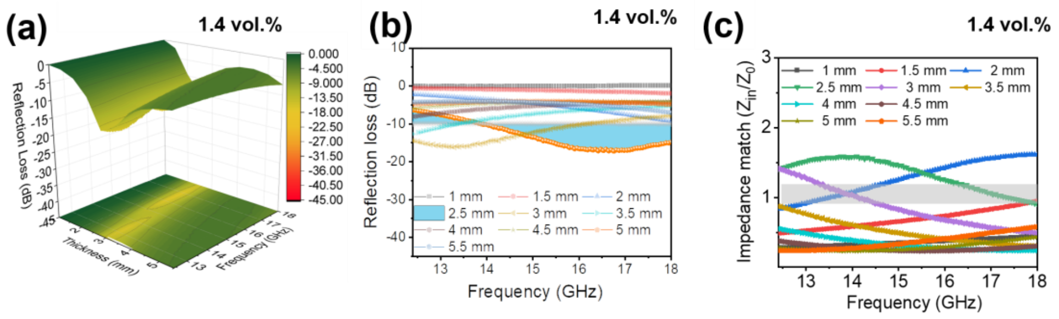

Fig. S9 3D EMW absorption micrographs (**a**), 2D EMW absorption micrographs (**b**) and impedance match (Z_{in}/Z_0, c) of PVTMS@MWCNT nano-aerogel (filler content: 1.4 vol.%)

Fig. **S9**a, b show the EMW absorption property of PVTMS@MWCNT nano-aerogel with 1.4 vol.% nanofiller content, and **Fig.** S9c shows the impedance match of the sample. However, due to the low attenuation constant of PVTMS@MWCNT nano-aerogel (**Fig.** 3d), hence the PVTMS@MWCNT nano-aerogel shows low EMW absorption properties.

S1.4 IR Stealth Property of PVTMS@MWCNT Aerogel with Various MWCNT Content

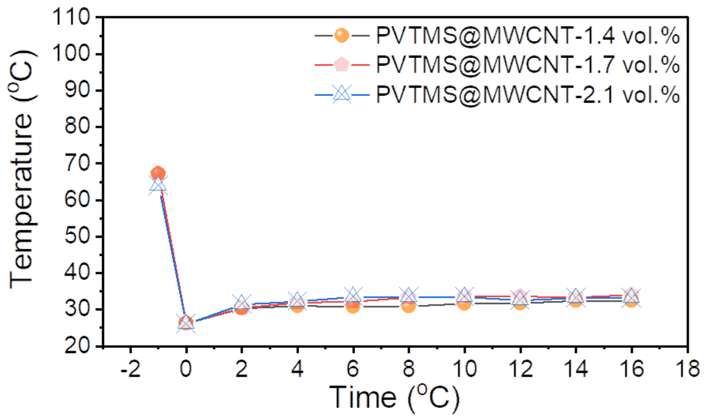

Fig. S10 Variation tendency of the temperature detected on the upper surface of PVTMS@MWCNT aerogel with various MWCNT content

Fig. **S10** shows the IR stealth property of PVTMS@MWCNT aerogel with various MWCNT content, it is noted that the IR stealth property could be maintained with increasing MWCNT nanofiller content. This could be ascribed to the nanopore size generated in the nano-aerogel system greatly decreasing the thermal conduction.

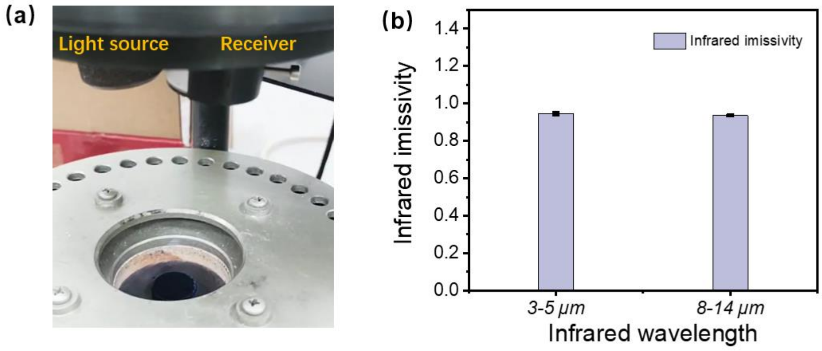

Fig. S11 Digital photo for infrared emissivity test (**a**); infrared emissivity results for PVTMS@MWCNT-1.7 vol.% (**b**)

It is well known that all matter with a temperature above absolute zero constantly emits thermal radiation (infrared radiation). Infrared waves cover the range of 0.76 to 1000 μm and can be sub-divided into five parts: Near-infrared (0.76–1.5 μm), Short-wave infrared (1.5–3 μm), Medium-wave infrared (3–8 μm), Long-wave infrared (8–15 μm) and Far-infrared (15–1000 μm). It is worth noting that the Earth's atmosphere absorbs most of the infrared and is only relatively transparent to electromagnetic waves in the 3–5 and 8–14 μm range. Therefore, infrared emissivity in the 3–5 and 8–14 μm range are important for IR stealth application [S3].

As Fig. S11a, b shows, infrared emissivity results for PVTMS@MWCNT-1.7 vol% at 3–5 and 8–14 μm range were tested. It is noted that our PVTMS@MWCNT samples shows high infrared emissivity (0.95 at 3–5 μm and 0.94 at 8–14 μm). The proposed reason for the high infrared emissivity could be ascribed to the added MWCNT, which could act as black body, hence to absorb and emit IR signal.

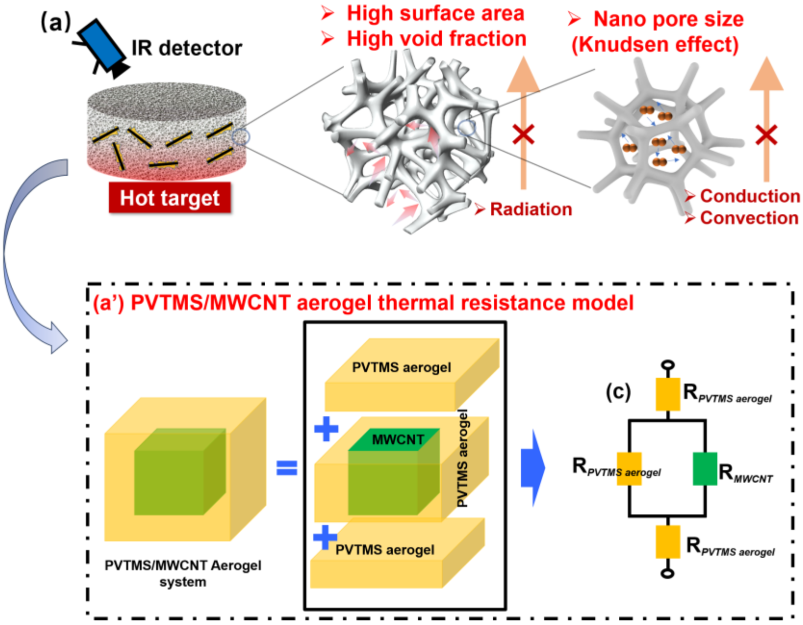

Fig. S12 Thermal conduction model for PVTMS@MWCNT nano-aerogel (**a**)

As Fig. 4c shows, the PVTMS@MWCNT nano aerogel in this work could greatly suppress the heat transfer from hot target to the upper surface. For PVTMS@MWCNT nano aerogel, it could be divided into two parts: (1) ultra-low thermal conduction part (PVTMS nano-aerogel); (2) high thermal conductivity part (MWCNTs);

As Fig. 2d shows, PVTMS nano-aerogel structure shows even lower thermal conductivity than air (26 mW·m^{-1} K^{-1}). Therefore, as Fig. S12c shows, PVTMS with low thermal conductivity could greatly enhance the overall thermal resistance of PVTMS@MWCNT system [S4, S5].

Supplementary References

[S1] J. Liu, F. Zhao, Q. Tao, J. Cao, Y. Yu et al., Visualized simulation for the nanostructure design of flexible strain sensors: From a numerical model to experimental verification. Mater. Horiz. **6**, 1892-1898 (2019). https://doi.org/10.1039/C9MH00389D

[S2] S. Wang, Y. Huang, C. Zhao, E. Chang, A. Ameli et al., Theoretical modeling and experimental verification of percolation threshold with mwcnts' rotation and translation around a growing bubble in conductive polymer composite foams. Compos. Sci. Technol. **199**, 108345 (2020). https://doi.org/https://doi.org/10.1016/j.compscitech.2020.108345

[S3] J. Hu, Y. Hu, Y. Ye, R. Shen, Unique applications of carbon materials in infrared stealth: A review. Chem. Eng. J. **452**, 139147 (2023). https://doi.org/https://doi.org/10.1016/j.cej.2022.139147

[S4] P. Buahom, C. Wang, M. Alshrah, G. Wang, P. Gong et al., Wrong expectation of superinsulation behavior from largely-expanded nanocellular foams. Nanoscale **12**, 13064-13085 (2020). https://doi.org/10.1039/d0nr01927e

[S5] G. Wang, C. Wang, J. Zhao, G. Wang, C. B. Park et al., Modelling of thermal transport through a nanocellular polymer foam: Toward the generation of a new superinsulating material. Nanoscale **9**, 5996-6009 (2017). https://doi.org/10.1039/c7nr00327g
